# Opportunities and Limitations of a Gaze-Contingent Display to Simulate Visual Field Loss in Driving Simulator Studies

**DOI:** 10.3389/fnrgo.2022.916169

**Published:** 2022-06-10

**Authors:** Bianca Biebl, Elena Arcidiacono, Severin Kacianka, Jochem W. Rieger, Klaus Bengler

**Affiliations:** ^1^Chair of Ergonomics, School of Engineering and Design, Technical University of Munich, Garching, Germany; ^2^Chair of Software and Systems Engineering, Department of Informatics, Technical University of Munich, Garching, Germany; ^3^Department of Psychology, University of Oldenburg, Oldenburg, Germany

**Keywords:** visual field loss, simulation, driving, gaze-contingent display, compensation, scanning

## Abstract

**Background:**

Research on task performance under visual field loss is often limited due to small and heterogenous samples. Simulations of visual impairments hold the potential to account for many of those challenges. Digitally altered pictures, glasses, and contact lenses with partial occlusions have been used in the past. One of the most promising methods is the use of a gaze-contingent display that occludes parts of the visual field according to the current gaze position. In this study, the gaze-contingent paradigm was implemented in a static driving simulator to simulate visual field loss and to evaluate parallels in the resulting driving and gaze behavior in comparison to patients.

**Methods:**

The sample comprised 15 participants without visual impairment. All the subjects performed three drives: with full vision, simulated left-sided homonymous hemianopia, and simulated right-sided homonymous hemianopia, respectively. During each drive, the participants drove through an urban environment where they had to maneuver through intersections by crossing straight ahead, turning left, and turning right.

**Results:**

The subjects reported reduced safety and increased workload levels during simulated visual field loss, which was reflected in reduced lane position stability and greater absence of large gaze movements. Initial compensatory strategies could be found concerning a dislocated gaze position and a distorted fixation ratio toward the blind side, which was more pronounced for right-sided visual field loss. During left-sided visual field loss, the participants showed a smaller horizontal range of gaze positions, longer fixation durations, and smaller saccadic amplitudes compared to right-sided homonymous hemianopia and, more distinctively, compared to normal vision.

**Conclusion:**

The results largely mirror reports from driving and visual search tasks under simulated and pathological homonymous hemianopia concerning driving and scanning challenges, initially adopted compensatory strategies, and driving safety. This supports the notion that gaze-contingent displays can be a useful addendum to driving simulator research with visual impairments if the results are interpreted considering methodological limitations and inherent differences to the pathological impairment.

## Introduction

### Theoretical Background

The prevalence of age-related diseases, such as visual impairments, will increase in the future due to demographic change. Wu et al. (2018) estimate that the number of persons affected by age-related macular degeneration alone will experience a worldwide growth from 196 million in 2020 to 288 million in 2040. Sufficient spontaneous recovery of visual field loss (VFL) is seldom, which implies persistent visual impairments in many cases (Zhang et al., 2006; Schuett et al., 2009a). Studies have found affected persons to exhibit increased exploration times and unsystematic scanning patterns in visual search tasks (Schuett et al., 2009a). In the context of driving, visual exploration is specifically challenging due to the high number of unnaturally fast-moving objects (Pomarjanschi et al., 2013), which results in reduced safety levels and increased crash involvement among some drivers with VFL (Johnson and Keltner, 1983; Haymes et al., 2008; Kwon et al., 2016; Biebl et al., 2021). While regarded as highly beneficial for driving safety, the ability to develop appropriate compensatory strategies shows high heterogeneity among individuals with VFL (Bowers et al., 2009; Elgin et al., 2010; Hardiess et al., 2010; Wood et al., 2011; Papageorgiou et al., 2012; Haan et al., 2014; Bahnemann et al., 2015; Kübler et al., 2015; Alberti et al., 2017). The great variance in driving performance among affected drivers indicates the existence of additional influencing factors that determine the ability to adopt behavior appropriately. However, it is currently unclear which personal and contextual determinants play a relevant role (Zihl, 1995; Tant et al., 2002; Patterson et al., 2019). In this context, research on driving with visual impairments poses multiple challenges that aggravate the systematic evaluation and identification of underlying mechanisms. One approach to account for these difficulties is the simulation of VFL. Prior research on simulated VFL has shown a wide variety of use cases and methodological approaches, which are summarized in the following two subchapters.

### Use Cases for Simulating Visual Field Loss

Medical simulations are generally defined as a “person, device, or set of conditions, which attempts to present evaluation problems authentically” (McGaghie and Issenberg, 1999). In addition to enriching research on visual impairments, simulations can be used for demonstration purposes. Reina et al. (2011), for example, used a multistage intervention program to increase children's awareness of visually impaired peers and found that a longer program, including the simulated experience of the impairment, led to better results than a shorter program without the simulation. Demonstrating a visual impairment can, therefore, help unaffected individuals understand its impact and help those affected to recognize the onset of functional degeneration in its early stages and anticipate the progression of the disease (Crabb et al., 2013). A hands-on experience of deficits is also crucial for clinicians to ensure accurate diagnostic classification of patients' descriptions. Therefore, simulations of VFL could also be beneficial in the education of medical students (Crabb et al., 2013; Juniat et al., 2019). Experiencing the impact of VFL on everyday tasks and the associated frustration and social isolation could, furthermore, improve clinician-patient relationships by increasing empathy and communication, and thereby improving outcomes and patient satisfaction (Crabb et al., 2013; Goodman-Deane et al., 2013; Juniat et al., 2019).

The simulation of VFL can, furthermore, serve as a fruitful tool for rehabilitation specialists or product designers to develop assistive technologies specifically designed to support persons with visual impairments. Interacting with users to identify their needs as part of the design process can be tedious, since users often have difficulty expressing their experiences with a product and, in many cases, lack the technical understanding necessary to develop new ideas (Kamikubo et al., 2017; Raviselvam et al., 2022). Simulations can improve the designers' understanding of user needs and improve the design process, especially in the early stages (Väyrynen et al., 2016; Kamikubo et al., 2017). Researchers in the past have already used simulations of impairments to identify and tackle problem areas of current technologies. Noteworthy mentions are Ford's so-called *third age suit* for a simulation of motoric, sensory, and visual age-related impairments during vehicle usage and approaches, focusing on way finding or medication management difficulties (Zagar and Baggarly, 2010; Rousek and Hallbeck, 2011; Raviselvam et al., 2022). Innovative concepts tend to receive better scores concerning novelty, quantity, and breadth if they are developed after experiencing the simulated impairment compared to theoretical explanations (Raviselvam et al., 2022).

### Simulation Methods

The strengths and limitations of different simulation techniques will be presented in the following section with a focus on the simulation of visual field defects. However, it should be pointed out that, while currently available, simulation methods can provide valuable insights, they cannot replace the interaction with, and investigation of, persons exhibiting actual deficits during research and product development (Jones et al., 2020; Macnamara et al., 2021).

A very cost-effective and easy-to-implement method is the digital alteration of pictures, where parts of a fictive or photographic scene are masked or cut out. This method can be useful for early stages of research and product development or general demonstration purposes but is too simplified to allow for an actual experience of the VFL's characteristics. Users do not actively engage with the static blurred or blind part of the scene but, rather, can look around it (Jones et al., 2020; Macnamara et al., 2021).

The utilization of contact lenses counteracts this issue with a fixed opaque spot on a contact lens that moves with the eye. In theory, contact lenses allow free movement and longer-term exposure to the VFL during everyday activities without the constraints of a desk setup or computer screen (Czoski-Murray et al., 2009). However, the challenging implementation limits this versatility. They can be uncomfortable for some subjects, depending on the type of contact lens, and special hygiene measures must be considered to avoid corneal infections (Robertson and Cavanagh, 2008). An approach by Foley-Fisher and Murphy (1987) used a tight-fitting scleral contact lens with an attached aluminum-alloy tube containing a black card to simulate scotoma. This approach was part of an intricate setup that did not allow users to walk around freely. A more versatile option is the use of soft contact lenses. However, this approach requires a constant and standardized pupil dilation to ensure a stable and comparable scotoma size (Nau, 2012; Butt et al., 2015) by the use of appropriately bright illumination or miotic drugs (Czoski-Murray et al., 2009; Klee et al., 2018). Miotic drugs are intrusive and not applicable for use cases where accommodation to different target depths is required. Constant bright lighting, on the other hand, may not be viable for use cases with specific lighting requirements, such as the darkened vehicle interior in a driving simulator. Almutleb et al. (2018) reported positive effects when using contact lenses with an opaque center. The authors pointed out that central positioning and retinal stabilization, contact lens rotation, and pupil size fluctuations must be considered in addition to implementation in a bright surrounding with miotic drugs. Furthermore, the size of the relative VFL introduced with centrally opaque contact lenses may vary due to the parallax effect, in which a large portion of the visual field is darkened and blurry with a smooth transition to the darkest spot in the center (Butt et al., 2015; Almutleb et al., 2018). This does not reflect reports by patients with age-related macular degeneration. At the current time, most studies using contact lenses have focused on central vision loss. Klee et al. (2018) presented an approach with an adjustable scotoma size. More complex forms of VFL with multiple blind spots may, however, not be viable (Czoski-Murray et al., 2009). Reports on simulations of peripheral vision have also been scarce. While theoretically possible, Nau (2012) did not find promising results with their implementation. Due to the restrictions concerning VFL presentation and implementation with contact lenses, this approach may not be suitable for research in a driving simulator.

One popular approach is the utilization of partially occluded glasses. Such glasses are non-intrusive and allow for free movement during the presentation of dynamic scenes. Implementation is often time- and cost-effective by applying paint or occlusion tape on glasses, goggles, or lenses in trial frames or by purchasing appropriate products, such as shutter glasses (Maiello et al., 2018; Swan et al., 2020; Macnamara et al., 2021). Most studies using this method for the simulation of visual impairments have focused on refractive blur or cataract simulations with an overall reduction of visual acuity and contrast sensitivity to investigate eye-hand coupling, speech-reading abilities, reading performance, and cognitive performance in different age groups (Bowers and Reid, 1997; Wood et al., 2009, 2010; Dickinson and Taylor, 2011; Goodman-Deane et al., 2013; Maiello et al., 2018; Swan et al., 2020). In regard to driving, adapted glasses have been used in closed-course tracks (Higgins and Wood, 2005; Wood et al., 2010), driving simulator studies (Lehsing et al., 2019), and hazard-detection videos (Lee et al., 2016). Generally, research with low vision simulator goggles has revealed promising results, whereas simulations of VFL with this method have been scarce. Brooks et al. (2005) implemented monocular tunnel vision by occluding one eye and applying a cone with a small opening in front of the other eye in a driving simulator. The monocular presentation of the VFL prohibited blurred vision that can arise from occluding different parts of the binocular field of view when masking each eye individually with altered glasses (Rousek and Hallbeck, 2011; Lee and Itoh, 2020). Another problem when simulating VFL with glasses is the option for users to look past the occluded parts by moving their eyes. While useful for initial educational purposes (Aballéa and Tsuchiya, 2007), this may limit the generalizability of results from driving studies with simulated VFL.

Wood et al. (2010) proposed using so-called gaze-contingent displays (GCDs) to counteract the mentioned limitations of other simulation techniques. The gaze-contingent paradigm describes the continuous change of a display (e.g., concerning partial resolution degradation, masking of objects, or appearance of stimuli) in accordance with the current gaze position. Researchers have also used the descriptions moving mask technique (Rayner and Bertera, 1979) and moving window technique (Duchowski et al., 2004) for degradation of foveal and peripheral information, respectively. Aside from research, GCDs have found applications in product design. Tobii eye trackers, for example, are designed to enhance video games by replacing a hand-held cursor with a gaze-dependent cursor and by adapting lighting and avatar direction within the video game according to gaze direction. Breuninger et al. (2011) implemented a gaze-contingent device operation for users suffering from tetraplegia. Padmanaban et al. (2017) presented an adaptive focus display to counteract myopia and hyperopia. The gaze-contingent paradigm is also applied in screens with a complex graphical user interface (e.g., virtual reality) to decrease rendering time by peripheral resolution degradation (Duchowski et al., 2004). Gaze-contingent visual degradation, especially in the peripheral visual field, has also been used to investigate the role of central and peripheral vision in reading, navigation, and visual search (Perry and Geisler, 2002; Geisler et al., 2006; Castelhano and Henderson, 2007; Lingnau et al., 2008; Võ and Henderson, 2010). The gaze-contingent approach in these studies eliminates the restrictions of prior single-fixation approaches to enable a more natural gaze behavior during visual search or UFOV tasks (Geisler et al., 2006; Ringer et al., 2015; Gaspar et al., 2016). The presentation of additional gaze-contingent stimuli has also been used in research on driver assistance systems, where directional cues or enhanced-vision systems highlight and attract the driver's attention toward relevant areas (Pomarjanschi et al., 2012, 2013; Wade et al., 2016). Lastly, GCDs have served as a valuable tool to simulate VFL. Implementation of virtual reality software has enabled the investigation of problems for subjects with tunnel vision during everyday tasks, such as mobile phone localization (Jones et al., 2020) and navigation through a city (Väyrynen et al., 2016) or the identification of deficiencies in the design of escape-route signs for homes for the elderly (Krösl et al., 2018). Screen-based GCD implementations have been used to simulate central scotoma, with research focusing on visual exploration, object perception, and reading in age-related macular degeneration (Henderson et al., 1997; van Diepen et al., 1998; Walsh and Liu, 2014) and tunnel vision (Rayner and Bertera, 1979; Castelhano and Henderson, 2007; Võ and Henderson, 2010), as well as rapid prototyping (Kamikubo et al., 2017) under complete occlusion of the peripheral field of view. Furthermore, GCDs have been used to simulate peripheral VFL, resembling homonymous hemianopia. Simpson et al. (2011) simulated left-sided and right-sided hemianopia in healthy participants and compared the results of a visual search task to reports from pathological hemianopia. They found longer scan paths and a shifted spatial distribution to the blind side, which matches reports from other studies on simulated (Tant et al., 2002; Machner et al., 2009; Schuett et al., 2009a,b) and (at least early) pathological hemianopia (Zihl, 1995; Pambakian et al., 2000; Tant et al., 2002; Machner et al., 2009). Tant et al. (2002) investigated performance in a dot-counting task under simulated and pathological hemianopia and found more fixations within the blind field and smaller saccades into the blind field for both groups. While demonstrating parallel trends for simulated and pathological VFL in many parameters, the simulated homonymous VFL seemed to have a greater impact on visual search (Tant et al., 2002). Nowakowska et al. (2016, 2019) conducted two studies with simulated hemianopia in a visual search task. They found that simulated hemianopia resembled pathological hemianopia in their tendency to make a first scan to the blind side. A subsequent scanning bias toward the blind side typically reported for pathological homonymous hemianopia, which was only evident in the second study. This shows that the interpersonal heterogeneity in scanning behavior and the varying degrees of spontaneously developed compensatory strategies cannot only be found in patients but also upon immediate confrontation with a simulated VFL (Nowakowska et al., 2016, 2019).

### Scope

Overall, research on the utilization of GCDs to simulate different types of VFL has yielded promising results when compared to the effect of the corresponding pathological deficit on task performance. It has, furthermore, been proposed as the best currently available approach to simulate VFL. Research on the applicability of GCDs to simulate VFL in the context of driving has been scarce. Glen et al. (2015, 2016) conducted two studies investigating the impact of different upper or lower VFLs (2015) and a leftward or rightward scotoma (2016) on driving-related hazard perception test performance. Results suggested a reduction of hazard test performance with simulated VFL that parallels findings of patients with glaucoma (Crabb et al., 2010; Lee et al., 2017). To the authors' knowledge, no research has yet been conducted using simulated VFL with the gaze-contingent paradigm in a high-fidelity driving simulator where participants must actively maneuver a vehicle through a driving scene spanning a field of view that includes far peripheral vision. The implementation of simulated homonymous hemianopia in such a setup and results from a first study to evaluate its effect on driving and visual behavior is reported in the following chapters. Findings from this study are then referenced to existing reports on pathological homonymous hemianopia as the basis for a general discussion on the opportunities and limitations of GCDs to simulate VFL in driving simulations.

## Materials and Methods

### Apparatus

**Figure 1:**
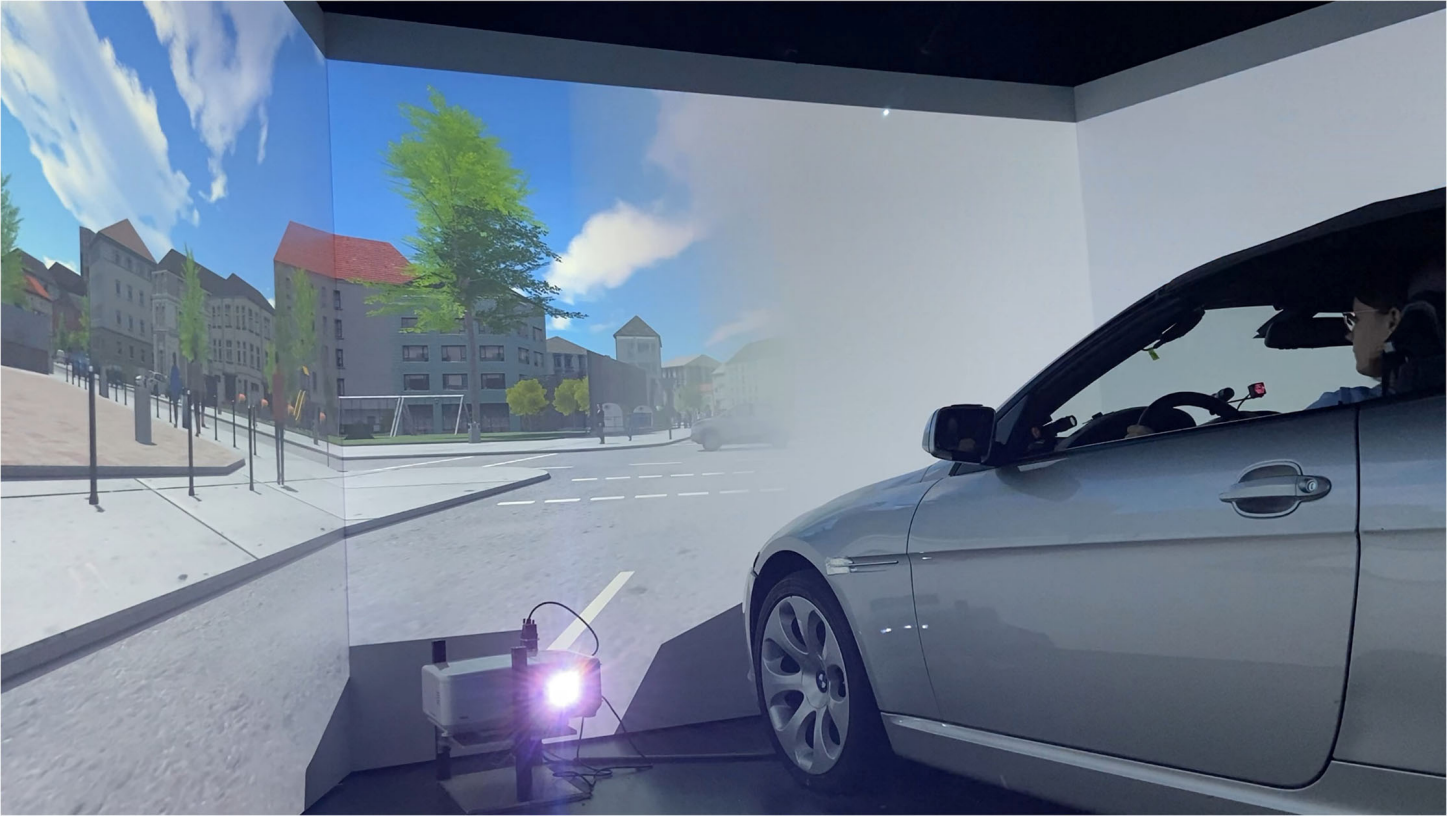
The study was conducted in a static driving simulator, which consists of a BMW mock-up and a 180° frontal field of view. Complete homonymous hemianopia on the left and right side was simulated on large screens with a semi-transparent transition toward the seeing side and a match of color and luminance of the occluding mask to the road surface and surrounding housing.

The simulation was implemented, and the study was conducted in the static driving simulator of the Chair of Ergonomics at the Technical University of Munich (see Figure 1). The driving simulator consisted of a BMW E64 vehicle mock-up that allowed the pedals and steering wheel to be operated as in a normal vehicle with no simulation of driving dynamics through vestibular feedback. The vehicle was surrounded by three large screens, each spanning a size of 3,400 x 2,720 pixels and constituting a total 180° front view. Three additional screens presented the rear view. All screens received visual input from projectors with a refresh rate of 60 Hz. The driving scenery was simulated with SILAB 6.5 of the Würzburg Institute for Traffic Sciences GmbH (2020) with a refresh rate of 60 Hz. Driving data were collected with a rate of 240 Hz. A six-channel sound simulation was implemented in the vehicle mockup. Eye and head tracking was performed *via* three stationary SmartEye Pro cameras with software version 8.0 installed in the interior of the vehicle mock-up. Eye tracking data were collected with a rate of 60 Hz. SILAB and SmartEye Pro ran on two separate computers connected *via* Ethernet.

### VFL Simulation

For simulating the VFL, we used the interface between SmartEye Pro and SILAB to continuously send the current gaze position to the driving simulator software. Gaze position was operationalized with the variable *GazeDirection* provided by SmartEye Pro, which represents a “unit vector originating in the virtual eye position, describing the direction of the gaze” (*SmartEye Pro*, 2018). X- and y-coordinates of the gaze direction were used as input variables for a ruby script, which computed the respective coordinates within the SILAB coordinate system. The three adjacent projections of the driving scene were controlled by different computers, so the coordinate systems' transformation was performed separately for each processor. The three resulting coordinate points in the SILAB coordinate systems served as the anchor position for a 2D-overlay that masked either the complete left or right side with regard to the current gaze position. This process was repeated continuously with a frequency of 60 Hz.

The driving simulator environment introduces certain challenges concerning implementation compared to previous setups. Most screen-based GCDs have used a desk-mounted monitor with a maximum horizontal field of view of 60° or less (Simpson et al., 2011; Walsh and Liu, 2014; Wu et al., 2018). For investigating peripheral VFL, it is advisable to enable a field of view beyond 30 degrees on both sides, since this is often regarded as the beginning of the mid-periphery (Strasburger et al., 2011). A large field of view is especially crucial for driving to enable large gaze movements to detect potential hazards in the periphery. The frontal field of view of 180° in the driving simulator used for the present study fulfilled this requirement. Many studies using a GCD have used eye trackers with at least 120 Hz and, more often, up to a 1,000 Hz frame rate and/or a chin and head rest (Schuett et al., 2009a,b; Aguilar and Castet, 2011; Simpson et al., 2011; Walsh and Liu, 2014; Glen et al., 2015; Ringer et al., 2015; Kamikubo et al., 2017; Nowakowska et al., 2019). This optimizes eye tracking performance, which is one of the pivotal points for GCDs. However, head and chin rests prohibit large gaze movements toward the periphery since head movements usually accommodate gaze shifts above an amplitude of ~20 degrees (Stahl, 1999). Such constraints are, therefore, not viable for scanning a driving scene, particularly at intersections. Reduced eye tracking performance during large head scans in the current setup was accounted for by using the last available data point in case of signal loss. A moving window buffer was included to counteract potential signal instability. While this can increase system latency, it is not known exactly how latency between eye movements and a dislocation of the gaze-contingent mask affects task performance. As noted by Saunders and Woods (2014), system latency can stem from three sources: the eye tracker, the display, and the additional delays due to the operating system or interposed algorithms. The maximum tolerated delay that remains unnoticed and, more importantly, does not influence performance, however, is highly task dependent (Aguilar and Castet, 2011) and generally reduced for greater saccades (Loschky and Wolverton, 2007). For this reason, the buffer was eliminated for large gaze movements. One risk of a temporal mismatch between the gaze and mask position is that the user can see objects that were meant to be occluded. However, this risk is greater for smaller scotomas, especially in the central field, since a delayed relocation of the hemifield-spanning mask only leads to a prolonged occlusion of the target or earlier visibility of areas that were already visible with the unobscured peripheral visual field (Simpson et al., 2011).

ndividualization of the VFL can be performed easily by generating an image with a respective opaque field in an otherwise transparent area. Especially those studies investigating neurocognitive phenomena have used gaze-contingent masks with the same color and luminance as the background (Schuett et al., 2009a; Walsh and Liu, 2014; Nowakowska et al., 2016, 2019). This approach is useful to minimize visibility of the simulated blind visual field. It is reported that a more noticeable scotoma leads to enhanced gaze positioning as well as a facilitated and, therefore, faster, development of a compensatory-preferred retinal location (Sipatchin et al., 2021). We chose a gray mask that resembled color and luminance of the road and buildings within the simulated city (see Figure 1). A smooth, semitransparent fade between the masked and unmasked part of the visual scene was used to blur the mask's border. The GIMP graphics software version 2.10.30 was used to design the gaze-contingent masks, portraying complete left- or right-sided homonymous hemianopia. Homonymous hemianopia was chosen since it is one of the most frequent types of VFL after brain lesions (Suchoff et al., 2008; Matteo et al., 2016).

### Sample

The total sample size comprised 18 participants. Three participants had to be excluded because of an early study termination due to simulator sickness or insufficient calibration and, therefore, the performance of the eye tracker. The final sample consisted of 15 participants (5 females, 9 males, 1 diverse), with a mean age of 26.07 (*SD* = 9.60). The participants had no visual impairments except for near- or farsightedness corrected by glasses or contact lenses. They had held their driver's license for a mean of 8 years (*SD* = 9.39). Ten participants reported simulator sickness, with distinct hindering effects in four cases. Recruitment was carried out *via* university notices and by directly approaching potential candidates.

### Experimental Design

The study followed a 3-x-3within-subject design. Each participant completed three drives, without the VFL (normal vision; NV), with left-sided simulated homonymous hemianopia (LHH) or with right-sided simulated homonymous hemianopia (RHH). Within each drive, the participants experienced three intersection types where they had to either turn left (left-turning intersection; LTI), turn right (RTI) or cross straight ahead (straight-crossing intersection; SCI). The different maneuvers were used due to their varying demands to scan for potential hazards (Gerstenberger, 2015).

### Experimental Track

The experimental track consisted of an urban road map with straight roads, right and left curves, and intersections. The speed limit throughout the entire drive was 50 km/h (≅31 mph). All non-intersection portions were lined with parking vehicles, pedestrians, and general urban infrastructure on the sidewalk. There was oncoming traffic on the adjacent lane but no vehicle in their own lane that required attention or speed adaptation. The track included three intersections with two single-lane roads crossing at a 90° angle. A yield sign indicated that the drivers had to give the right of way, and a zebra crossing with the respective traffic sign indicated that pedestrians could cross on all four legs of the intersection. Pedestrians were present within the intersection area but not close to the road or the zebra crossing so the drivers had to scan for vulnerable traffic participants but not interact with them. The drivers were required to yield to crossing traffic, with vehicles crossing the center of the intersection every 9 s. The three intersections were identical except for the required driving maneuver, which was indicated by a navigational arrow in the dashboard. The order of the intersections and the mid-block track was kept identical for all three drives with different vision conditions.

### Procedure

The participants received information about the study, gave their consent, and filled out a demographic questionnaire before visiting the driving simulator of the chair of ergonomics at the Technical University of Munich. Upon arrival, the participants received further information about the study, the simulated VFL, and the driving simulator. The participants then completed several basic fixation tasks within the driving simulator for a technical examination of the driving simulator and eye tracking system, which is not reported here. Before the drives, a four-point calibration of SmartEye Pro was executed. Calibration was repeated between drives if required, e.g., when the participants left and reentered the driving simulator. In a subsequent familiarization drive, the participants could get used to handling the driving simulator. To maximize familiarization with the type of maneuvers performed in the test drives, the identical track was used. However, traffic was excluded so that the participants could focus on operating the mock-up vehicle. The first test drive was always carried out under NV to allow for a comparable reference in the final interview after all the drives. The order of the following LHH and RHH drives was permuted to avoid sequence effects. To allow for familiarization with the simulated VFL and to avoid learning effects within the test drives, another LHH or RHH familiarization drive preceded the LHH and RHH test drives, respectively. Each test drive lasted between 5 and 7 min, and pauses were interposed if necessary. Pauses between drives were administered if necessary. Within the test drives, the participants were repeatedly asked to indicate their mental demand during the scenario they had just experienced. After all the drives, the participants answered interview questions about the simulated VFL and its corresponding difficulties, exerted compensatory strategies, and perceived overall safety. Overall, the entire appointment lasted about 75 min. Due to the ongoing COVID-19 pandemic, all surfaces used were disinfected after each participant, and the laboratory was ventilated for at least half an hour before welcoming the next participant.

The study protocol followed the constitutes of the Declaration of Helsinki and was approved by the Ethics Committee of the Technical University of Munich (reference No.: 439/21 S).

### Measures and Data Processing

Driving and visual behavior were measured continuously throughout the drives. A structured interview and a questionnaire executed after the drives provided subjective data on the driving experience. While objective and subjective measurements were taken for scenarios both with and without intersections, data analysis focused on intersections. It can be assumed that intersections are associated with a particularly high demand for hazard perception and scanning with large-gaze eccentricities and are, therefore, especially critical for drivers with peripheral VFL (Bowers et al., 2014; Biebl and Bengler, 2021). Three participants did not correctly perform the right-turn intersection with full vision and accidentally drove straight ahead. These data points were excluded from further analyses.

Concerning objective data, intersections were defined as the interval between first pressing the brake pedal and reaching the zebra crossing at the intersection. This is in accordance with the deceleration phase defined by Plavsic (2010). Since scanning and hazard detection are very crucial in this phase, it is particularly relevant for the evaluation of the effects of simulated visual impairments. Based on the approach by Bowers et al. (2014), brake activation was searched for within 100 m before the intersection. When the brake was not activated within this distance, the start of the considered interval was set to 41.76 m before the intersection, which conforms with the typical start of deceleration 3 s prior to reaching an intersection at a speed limit of 50 km/h (Plavsic, 2010).

Driving behavior can be divided into lateral and longitudinal guidance. The former was represented by the number of lane crossings, as well as the mean and variance of lane position. Lane position was defined by the offset to the ideal central lane position in centimeters, where positive values represented an offset to the right. Longitudinal guidance in the sense of speed or acceleration behavior was not analyzed *per se*, since the individual braking behavior when approaching an intersection does not allow for sensible group-wise comparisons of singular parameters. Instead, the duration of the considered deceleration phase (first brake to start of zebra crossing at intersection in seconds) was evaluated. This comprises the initial speed, distance to intersection when first braking, deceleration, and stopping time. Eye tracking data in the considered interval were processed in multiple steps before analyzing gaze behavior. Since horizontal gaze movements are of the most interest when evaluating the effect of simulated homonymous hemianopia, the horizontal gaze direction, which also served as an input variable for the GCDs, was used. Missing values in this variable due to signal loss were replaced by a less accurate but more stable gaze direction value based on head position. All blinks, gazes to the media system or other non-driving related areas and the mirrors were excluded from the analysis. The latter was necessary since the GCD only spanned the front view. A Butterworth filter with a low band of 10 Hz reduced noise. Gaze data were classified as a fixation if the horizontal position did not change more than 2 degrees within a 120-millisecond time window, and rotation velocity did not exceed 30 degrees per second. Saccades were defined as gaze movements with a minimum rotation velocity of 90 degrees per second. These values were based on the recommendation of DIN EN ISO 15007-2015 (DIN Deutsches Institut für Normung e. V. O., 2015). Analysis of visual behavior encompassed the side of the first large gaze (defined as a gaze position, with a minimum eccentricity of 30 or 45 degrees), mean number, and duration of fixations (in seconds) in the left and right hemifield (defined as the visual field beyond the central 10 degrees), mean horizontal amplitude of saccades (in degrees) to the periphery (defined as saccades going to the left with an endpoint eccentricity of more than 5 degrees into the left hemifield or *vice versa* for the right hemifield), the horizontal variance of gaze positions, as well as the minimum, maximum, and mean gaze positions within the considered interval (gaze position was expressed in degrees).

The subjects were asked to rate the perceived workload of each driving scenario on a 21-point scale (translated from German: “How much mental demand was required for perceiving and processing information to complete the driving task?”), resembling the mental demand item of the NASA-TLX. After all the drives, an interview was conducted to evaluate the occurrence of simulator sickness and the participants' experiences when driving with the simulated VFL. Questions included for data analysis targeted perceived challenges and adopted compensatory strategies regarding lateral guidance, longitudinal guidance, hazard perception, a general overview of the driving scene, and the interaction with other traffic participants. The participants were instructed to answer these questions for the simulated VFL compared to the drive with full vision to exclude the influence of interindividual problems, e.g., due to unfamiliarity with the driving simulator. Lastly, the participants rated their perceived overall safety levels on a scale from 0 to 100 for NV, LHH, and RHH.

Data analysis was performed descriptively and inferentially. Descriptive analyses were performed using Microsoft Excel 365 (Microsoft, 2021) and R Studio version 3.6.1. Data processing for all objective data and inferential analyses was performed using R. Descriptive analyses were executed by inspecting mean, standard deviation, and range of values as well as individual patterns. The latter was particularly relevant since a vast number of studies on drivers with pathological and simulated VFL have reported great interindividual differences in driving and visual behavior when confronted with a visual impairment (Bowers et al., 2009; Elgin et al., 2010; Hardiess et al., 2010; Wood et al., 2011; Papageorgiou et al., 2012; Haan et al., 2014; Bahnemann et al., 2015; Kübler et al., 2015; Alberti et al., 2017). We wanted to determine for each subject whether LHH or RHH induced identical, higher, or lower values compared to NV for driving and visual parameters. This was done by calculating the interquartile range of all the participants in the respective NV condition, evenly adding and subtracting half of this range around the subject's NV value and then comparing the subject's LHH and RHH value with the upper and lower bounds of this individual NV range, respectively:


$$\begin{array}{l} Value_{LHH/RHH}\in (Value_{NV}\;\mp (\frac{1}{2}\;\times (Value_{Sample\_NV\;(0.75)}\\ \;-Value_{Sample\_NV\;(0.25)}))) \end{array}$$

The subjects' LHH or RHH value was rated as equal, above, or below NV. According to this differentiation, this process will be referred to as the EAB analysis. For number of fixations, EAB analysis was also executed in a slightly modified form for the relation of values for the blind side compared to values for the unmasked (in the following: *seeing*) side for the simulated VFL conditions. EAB analysis was performed using Microsoft Excel 365. A full list of descriptive values can be found as Supplementary Materials online.

Inferential analyses were executed with a repeated measures ANOVA based on a mixed linear model with Satterthwaite's method in R's *lme4* package. The model used vision condition (NV, LHH, RHH) and intersection type (SCI, LTI, RTI) as fixed effects. For hemispace and directional analyses of fixation and saccades, the respective hemifield (left, right) was included as an additional fixed effect. Person effects were treated as random effects. *Post-hoc* pairwise comparisons were executed using Kenward-Roger methods for denominator degrees of freedom using the *emmeans* package. If required, alpha error adjustment was performed using Bonferroni correction.

## Results

### Driving Data

Mean lane position showed a general left-handed offset from the ideal lane position in all vision conditions, but was least pronounced during LHH (NV: *M* = −17.23; *SD* = 28.26; LHH: *M* = −7.35; *SD* = 24.78; RHH: *M* = −15.95; *SD* = 27.45). Inferential statistics showed no main effect of vision condition, *F*_(2, 108.95)_ = 2.03, *p* = 0.136, or intersection type, *F*_(2, 108.95)_ = 0.90, *p* = 0.412, and no interaction effect between both variables, *F*_(4, 108.95)_ = 1.04, *p* = 0.390. Viewing all EAB comparisons of the mean lane position offset compared to the intraindividual NV value in that intersection type, the participants shifted toward their seeing sides, allowing for a buffer on the blind side in 29.76% of cases. A greater buffer on the seeing side was collectively introduced in 22.62% of intersections with simulated VFL. When comparing simulated VFL, a buffer on the seeing side was less frequently observed during LHH compared to RHH (see Table 1).

**Table 1:** Number and percentage of intersections where the participants held a lane position that introduced a buffer on the blind or seeing side for both simulated VFL conditions.

		**LHH (*K =* 42)**	**RHH (*K =* 42)**
Buffer blind side	*k*	14	11
	*(%)*	(33.33%)	(26.19%)
Buffer seeing side	*k*	5	14
	*(%)*	(11.90%)	(33.33%)

Variance of lane position showed a descriptive increase in mean and range of values during NV (*M* = *0.6*6; *SD* = *0.4*7) compared to LHH (*M* = 2.12; *SD* = 3.48) and RHH (*M* = 2.16; *SD* = 3.81). Inferential analysis yielded no significant main effect of vision condition, *F*_(2, 108.65)_ = 1.79, *p* = 0.172, or intersection type, *F*_(2, 108.65)_ = 1.98, *p* = 0.143, and no interaction effect between both variables, *F*_(4, 108.63)_ = 0.38, *p* = 0.820. Three participants exhibited lane crossings in six of 132 recorded scenarios. All lane crossings happened during RHH (four cases) and LHH (two cases) drives.

The duration of the deceleration phase did not differ between conditions with an overall mean of 8.13 s (*SD* = 3.69) and a range between 1.06 and 20.15 s. Inferential analysis yielded no significant main effect of vision condition, *F*_(2, 109.16)_ = 0.91, *p* = 0.406, or intersection type, *F*_(2, 109.16)_ = 0.57, *p* = 0.570, and no interaction effect between both variables, *F*_(4, 109.15)_ = 0.64, *p* = 0.633. The distance to intersections when first activating the brakes served as the start of the measurement period for scenario duration and showed an overall large heterogeneity (range: [7.81; 99.93]; unit meters), including two cases with no brake activation when approaching SCI with full vision.

### Gaze Data

**Figure 2:**
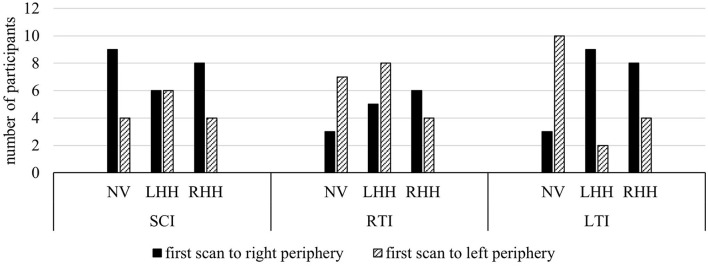
Number of participants making their first scan with a minimum amplitude of 30 degrees toward the left or right periphery. Compared to left-sided first scans, the first peripheral gaze movement was directed to the right hemifield by a similar or greater number of participants during SCI. For turning maneuvers, the participants were more prone to left-sided first scans with NV and right-sided first scans with RHH. LHH produced more right-sided first scans when turning left and more left-sided first scans when turning right.

A descriptive comparison of the side of the first peripheral scan showed that over all 132 intersection scenarios participants first scanned the right periphery in 43.18% of cases, the left periphery in 37.12% of cases, and no gaze to the periphery was made in 21.97% of cases. For SCI, the participants exhibited an equal or higher amount of first right-sided compared to left-sided gaze movements toward the periphery. For turning maneuvers, a difference between vision conditions was evident. Without VFL, the participants tended to first scan the left side, both when turning left and right. With RHH on the contrary, the participants were prone to first scan the right side in both turning intersections. With LHH, the participants tended to first scan the right side when turning left and to first scan the left side when turning right (see Figure 2). A comparison of blind and seeing side first scans for the simulated VFL showed that the first scan was more often directed to the blind side (48.89%; seeing side: 26.67%) with RHH and to the seeing side (44.44%; blind side: 35.56%) with LHH.

Some participants did not make any large gaze movement toward the periphery during the deceleration phase. For a description of missing peripheral scans, a criterion of 45° gaze eccentricity was used, representing the minimum required to perceive potential approaching hazards with an identical speed and, therefore, high collision risk during simulated VFL. In total, the participants did not make large gaze movements to the left periphery in 19.70% of intersections and to the right periphery in 32.57% of intersections. On both sides, more missing large scans were found for LHH (left periphery: 14; right periphery: 19) compared to NV (left periphery: 5; right periphery: 11) and RHH (left periphery: 7; right periphery: 13). Therefore, only LHH but not RHH produced an increased number of missing large scans on the blind as well as the seeing side. LHH, furthermore, showed a reduced mean of maximum gaze eccentricity on both sides (*M* = −43.97°|+ 42.57°; *SD* = 18.69°|19.95°) compared to NV (*M* = −55.57°|+51.60°; *SD* = 11.64°|15.47°) and RHH (*M* = −2.47°|+50.22°; *SD* = 15.24°|18.38°).

Analysis on horizontal gaze variance yielded a significant main effect of vision condition, *F*_(2, 109.08)_ = 7.65, *p* < 0.001, $n_{par}^{2}\;=$ 0.12. The main effect of intersection type, *F*_(2, 109.08)_ = 1.67, *p* = 0.192, and the interaction effect between both variables, *F*_(4, 109.07)_ = 0.57, *p* = 0.684, were not significant. *Post-hoc* pairwise comparisons showed that horizontal dispersion over all intersection types was significantly smaller for LHH compared to NV (△_Means_: −292.30°), *t*_(109)_ = −3.66, *p* = 0.001, *d* = −0.79, and RHH (△_Means_: −224.10°), *t*_(109)_ = −2.99, *p* = 0.010, *d* = −0.63. There was no significant difference between NH and RHH (△_Means_: −2.01°), *t*_(109)_ = 0.73, *p* > 0.999. Descriptively, mean horizontal gaze variance in RHH did not differ from NV during SCI (△_Means_: 6.32°) and LTI (△_Means_: 2.01°) but showed a smaller mean horizontal variance during RTI (△_Means_: 105.53°).

**Figure 3:**
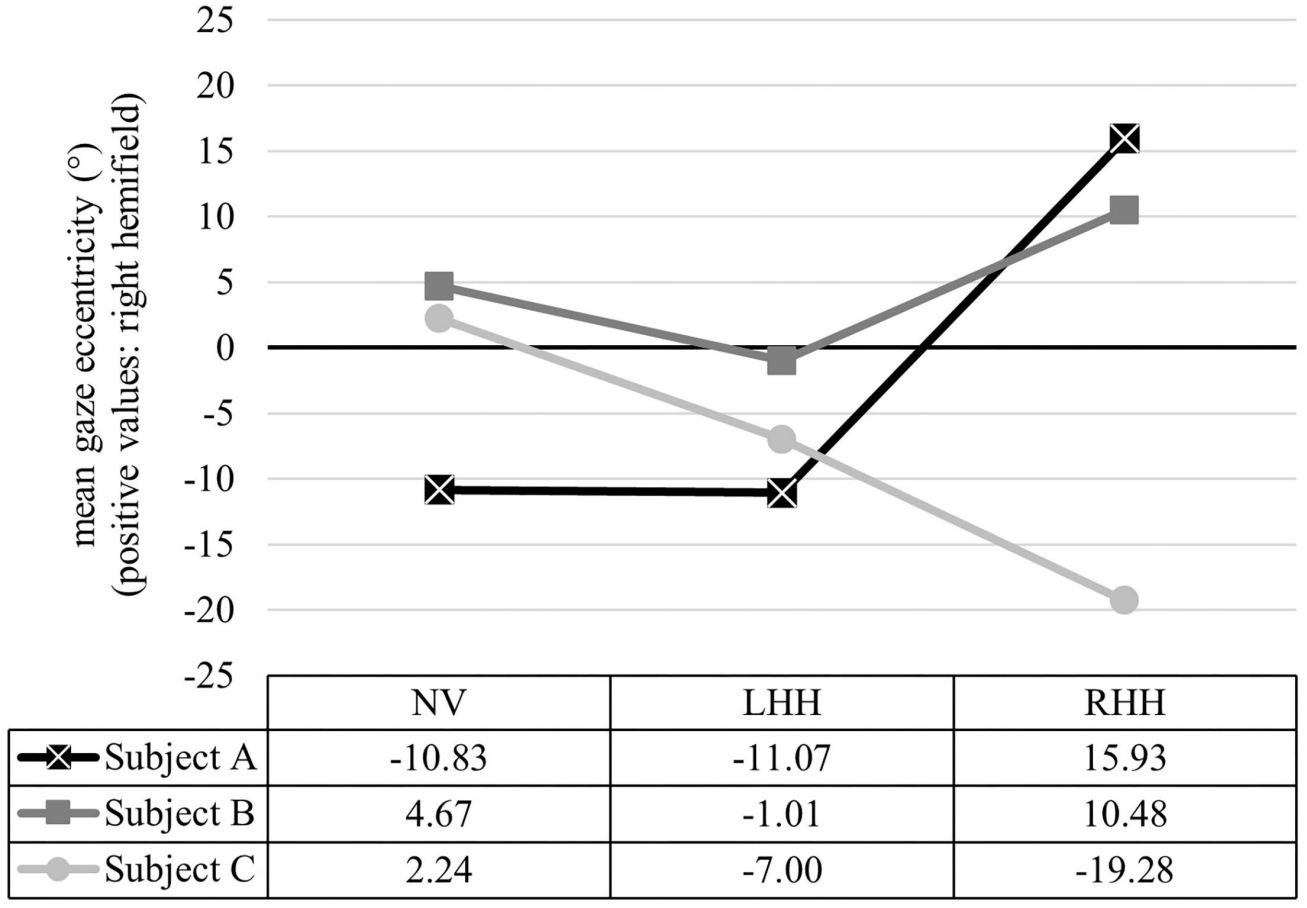
Exemplary data of mean gaze eccentricity for NH, LHH, and RHH for three participants. Relation of intersection conditions showed differing patterns between Subject A (NV = LHH < RHH), Subject B (LHH < NH < RHH), and Subject C (NV > LHH > RHH), reflecting great interindividual heterogeneity. Each data point represents the averaged mean gaze eccentricity over all intersection types.

Analysis on mean gaze eccentricity showed significant main effects of vision condition, *F*_(2, 109.58)_ = 3.11, *p* = 0.049, $n_{par}^{2}\;=$ 0.05, and intersection type, *F*_(2, 109.58)_ = 4.29, *p* = 0.016, $n_{par}^{2}\;=$ 0.07. The interaction effect was not significant, *F*_(4, 109.56)_ = 0.50, *p* = 0.737. *Post-hoc* pairwise comparisons yielded no significant differences between vision conditions. *Post-hoc* comparisons for intersection type showed that mean gaze eccentricity during RTI was further on the left than during LTI, with SCI descriptively ranking in between (see Table 2). Descriptively, gaze was directed more rightward with RHH compared to NH and LHH in all intersection types. For LHH, mean gaze position was further to the right compared to NV during LTI, but, otherwise, more to the left (see Table 3). Overall EAB analysis supported this pattern by showing that, during SCI and RTI, both simulated VFL conditions showed a shift toward the blind side in more cases (between 41.67 and 53.55%) than a shift toward the seeing side (between 20.00 and 33.33%) compared to NV. During LTI, however, both simulated vision conditions showed a higher percentage of shifts toward the right (53.33% for LHH and RHH) than the left (20.00% for LHH and RHH). On an individual level, a wide range of mean gaze eccentricities between −41.01° to the left and +38.00° to the right occurred, as well as various patterns with reversed effects of vision conditions (see Figure 3).

**Table 2:** Results of the *post-hoc* analysis for the main effects of vision condition and intersection type (^*^ indicates *p* < 0.05).

	**Vision condition**					**Intersection type**			
	***t***	***df***	***p***	***d***		***t***	***df***	***p***	***d***
NV—LHH	−0.27	110	>0.999	–	SCI—LTI	−0.34	109	>0.999	–
NV—RHH	−2.25	110	0.079	–	SCI—RTI	2.37	110	0.059	–
LHH—RHH	−2.03	109	0.134	–	LTI—RTI	2.70	110	0.024*	0.58

**Table 3:** Mean and standard deviation of mean gaze eccentricity per vision condition and intersection type.

		**NV**	**LHH**	**RHH**	**Ø**
SCI	*M*	0.33	−0.93	4.82	2.31
	*(SD)*	(7.06)	(15.51)	(11.14)	(1.22)
RTI	*M*	−6.41	−8.36	−0.27	−5.28
	*(SD)*	(8.31)	(17.93)	(15.81)	(1.33)
LTI	*M*	−2.18	3.51	5.54	3.08
	*(SD)*	(8.84)	(8.53)	(15.64)	(1.99)
Ø		−2.54	−1.93	3.36	
		(6.09)	(11.59)	(12.05)	

While mean gaze eccentricity can indicate a general dislocation of gaze position, analysis of fixations and saccades provides input for a hemispace analysis and a directional analysis (see Table 4 for descriptive values). This dissociation was already reported by Papageorgiou et al. (2012). For the hemispace analysis, mean number and mean duration of fixations were evaluated with Bonferroni correction. Mean number of fixations yielded a significant main effect of hemifield, with a higher number of fixations in the right than the left hemifield. All other main and interaction effects were not significant (see Table 5). When comparing vision conditions for each hemifield, the highest number of fixations in the left hemifield occurred with LHH and in the right hemifield with RHH. EAB analysis on number of fixations in the blind as compared to the seeing side showed that the participants made a higher number of fixations on the blind than on the seeing side in more cases (42.22%) than *vice versa* (28.89%). Such an intraindividual outnumbering of the blind side compared to seeing side fixations was found distinctly more often with RHH (55.56%) than LHH (28.89%). On the other hand, a higher number of seeing side compared to blind side scans was, generally, more seldom but could be found more often with LHH (35.56%) than RHH (22.22%).

**Table 4:** Mean and standard deviation of mean fixation number, fixation duration, and saccadic amplitude per vision condition and hemifield.

		**Hemispace analysis**				**Directional analysis**	
		**In left hemifield**		**In right hemifield**		**To left hemifield**	**To right hemifield**
		**Fixation number**	**Fixation duration (s)**	**Fixation number**	**Fixation duration (s)**	**Saccadic amplitude (**°**)**	**Saccadic amplitude (**°**)**
NV	*M*	2.78	246.45	4.18	266.34	32.06	27.28
	*(SD)*	(2.45)	(75.37)	(2.79)	(77.97)	(9.84)	(8.31)
LHH	*M*	3.67	318.97	3.98	350.89	19.60	20.52
	*(SD)*	(2.58)	(150.57)	(2.33)	(158.54)	(10.37)	(9.28)
RHH	*M*	2.56	307.43	5.29	317.06	29.45	26.74
	*(SD)*	(2.85)	(73.45)	(3.92)	(119.83)	(14.08)	(18.43)

**Table 5:** Results of the mixed-model ANOVA for number of fixations (Bonferroni corrected for additional analysis on fixation duration, see Table 6).

		**Number of fixations**			
	***F***	***df1***	***df2***	***p***	** $\eta_{par}^{2}$ **
Vision condition	0.69	2	232.31	>0.999	–
Intersection type	0.90	2	232.31	0.814	–
Hemifield	14.00	1	232.05	<0.001	0.06
Vision condition * intersection type	0.64	4	232,29	>0.999	–
Vision condition * hemifield	3.55	2	232.05	0.061	–
Intersection type * hemifield	1.72	2	232.05	0.362	–
Vision condition * intersection type * hemifield	1.27	4	232.05	0.567	–

An absence of fixations in the left hemifield was found in 22.72% of all 132 cases (by 11 subjects) and in 12.12% of cases (by seven subjects) for the right hemifield. Most missing fixations were found with LHH for both hemifields, and most participants without fixations in one condition had missing fixations in multiple conditions. Missing fixations in both hemifields were found in six cases, four of which occurred with LHH. Individual differences of fixation distribution could be seen. One participant, for example, made no left-sided fixations in six of nine conditions but up to 12 fixations in the right hemifield in these situations. Overall, there were no clear trends that support the assumption that missing fixations resulted from increased gaze behavior in the opposite hemifield.

Bonferroni corrected analysis on mean duration of fixations in the left and right hemifields yielded a significant main effect of vision condition. All other main and interaction effects were not significant (see Table 6). *Post-hoc* pairwise comparisons showed a higher fixation duration with LHH compared to NV, *t*_(189)_ = 3.06, *p* = 0.015, *d* = 0.52. Descriptively, RHH ranked in between but showed no significant difference to LHH, *t*_(189)_ = −1.12, *p* > 0.999, or NV, *t*_(189)_ = 2.01, *p* = 0.274. On a descriptive level, fixation duration was longer in the right than the left hemifield for all vision conditions, and NV had the shortest mean fixation duration compared to simulated VFL in both hemifields.

**Table 6:** Results of the mixed-model ANOVA for fixation duration (Bonferroni corrected for additional analysis on number of fixations, see Table 5).

		**Number of fixations**			
	***F***	***df1***	***df2***	***p***	** $\eta_{par}^{2}$ **
Vision condition	4.83	2	188.17	0.018	0.05
Intersection type	0.69	2	187.78	>0.999	–
Hemifield	2.58	1	188.38	0.220	–
Vision condition * intersection type	0.19	4	187.76	>0.999	–
Vision condition * hemifield	1.02	2	187.48	0.727	–
Intersection type * hemifield	0.07	2	186.91	>0.999	–
Vision condition * intersection type * hemifield	0.14	4	187.41	>0.999	–

Directional analysis focused on mean saccadic amplitude with a significant main effect of vision condition. All other main and interaction effects were not significant (see Table 7). *Post-hoc* comparisons showed a significantly smaller mean saccadic amplitude in LHH than NV, *t*_(201)_ = −3.39, *p* = 0.003, *d* = −0.56. Descriptively, RHH ranked in between but did not show significant differences to LHH, *t*_(203)_ = 2.41, *p* = 0.051, or NV, *t*_(201)_ = −1.02, *p* = 0.931. Saccades toward the left periphery exhibited smaller mean amplitudes compared to saccades toward the right periphery in all vision conditions. Over all the intersection scenarios, mean saccadic amplitude ranged between 1.18° and 113.81°.

**Table 7:** Results of the mixed-model ANOVA for saccadic amplitude.

		**Saccadic amplitude**			
	***F***	***df1***	***df2***	***p***	** $\eta_{par}^{2}$ **
Vision condition	6.04	2	201.53	0.003	0.06
Intersection type	0.96	2	198.69	0.384	–
Hemifield	1.52	1	199.28	0.219	–
Vision condition * intersection type	0.29	4	198.76	0.886	–
Vision condition * hemifield	1.13	2	198.19	0.324	–
Intersection type * hemifield	0.77	2	197.04	0.463	–
Vision condition * intersection type * hemifield	0.29	4	197.10	0.881	–

### Qualitative Data

Overall safety was rated on a scale from 0 to 100. Table 8 shows the mean and standard deviation among vision conditions. Analysis revealed a significant main effect of vision condition, *F*_(2, 28)_ = 48.69, *p* < 0.001, $n_{par}^{2}\;=$ 0.78, and *post-hoc* pairwise comparisons showed higher subjective safety rankings for NV compared to LHH, *t*_(28)_ = 8.24, *p* < 0.001, *d* = 3.01 and RHH, *t*_(28)_ = 8.83, *p* < 0.001, *d* = 3.22. There was no significant difference between LHH and RHH, *t*_(28)_ = 0.59, *p* > 0.999.

**Table 8:** Mean and standard deviation of a subjective safety rating for each vision condition.

		**NV**	**LHH**	**RHH**
Subjective safety [1; 100]	*M*	84.00	42.00	39.00
	*(SD)*	(3.13)	(6.03)	(4.09)

The single-item workload estimation revealed significant main effects of vision condition, *F*_(2, 109.14)_ = 23.08, *p* < 0.001, $n_{par}^{2}\;=$ 0.30, and intersection type, *F*_(2, 109.14)_ = 3.77, *p* = 0.026, $n_{par}^{2}\;=$ 0.06. No interaction effect between both conditions was found, *F*_(4, 109.13)_ = 1.94, *p* = 0.007. *Post-hoc* pairwise comparisons showed higher workload values for LTI compared to SCI, *t*_(109)_ = 2.46, *p* = 0.047, *d* = 0.52. RTI descriptively ranked in between but did not show significant differences to LTI, *t*_(109)_ = −0.13, *p* > 0.999, *d* = −0.03, or SCI, *t*_(109)_ = 2.27, *p* = 0.075, *d* = 0.49. Concerning vision condition, NV received lower values than LHH, *t*_(109)_ = −6.31, *p* < 0.001, *d* = −1.36, and RHH, *t*_(109)_ = −5.45, *p* < 0.001, *d* = −1.18. There was no significant difference between LHH and RHH, *t*_(109)_ = 0.88, *p* > 0.999. Descriptively, turns toward the seeing side received the highest values for simulated VFL conditions (see Table 9).

**Table 9:** Mean and standard deviation of the single-item workload scale per vision condition and intersection type.

		**NV**	**LHH**	**RHH**	**Ø**
SCI	*M*	5.87	10.6	11.27	9.29
	*(SD)*	(3.48)	(3.91)	(4.70)	(0.80)
RTI	*M*	8.08	14.27	10.67	11.00
	*(SD)*	(3.96)	(5.38)	(4.20)	(0.84)
LTI	*M*	8.27	12.07	13	10.46
	*(SD)*	(4.54)	(4.11)	(5.54)	(1.17)
Ø		7.31	12.31	11.64	
		(3.42)	(4.01)	(4.10)	

**Figure 4:**
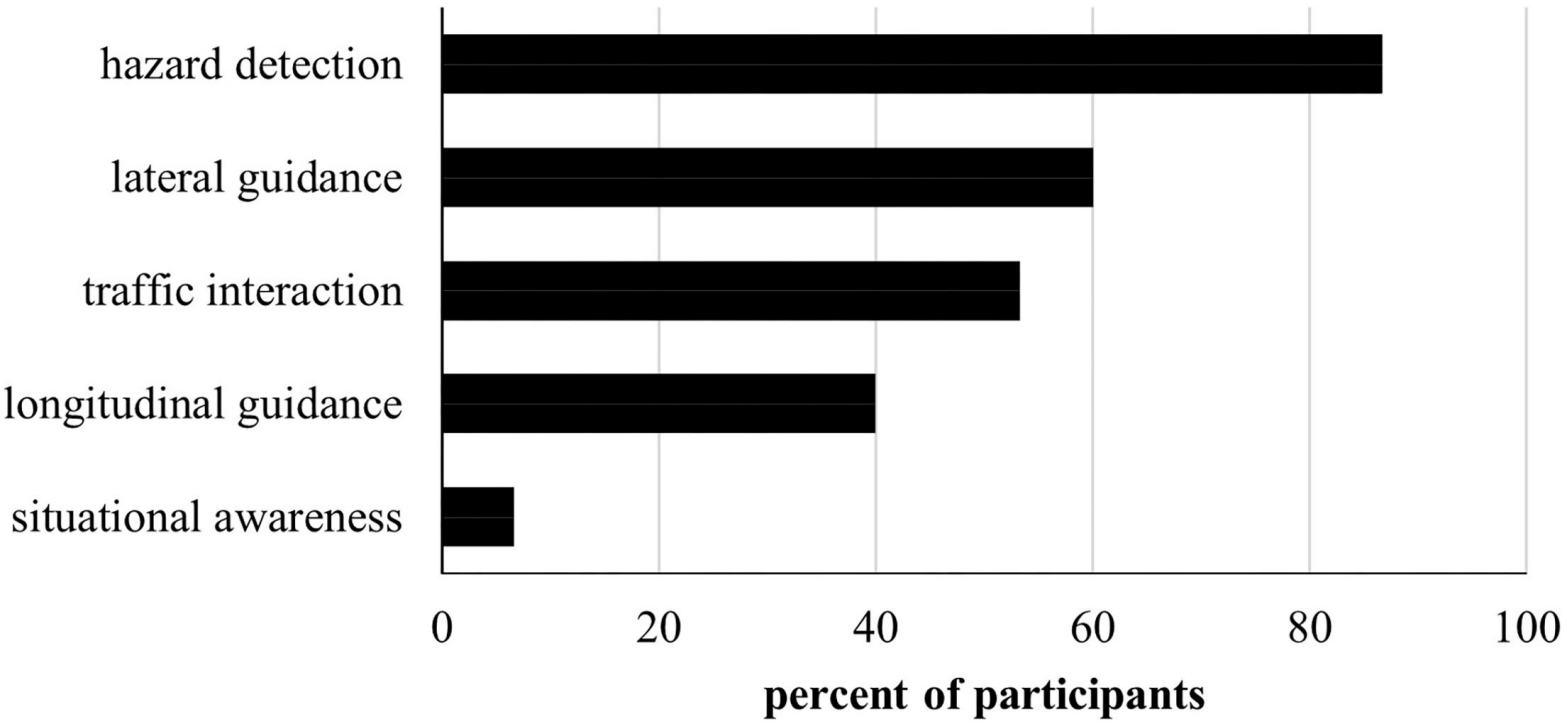
Number of participants confirming the experience of problems concerning hazard detection, lateral and longitudinal guidance, traffic interaction, and general situational awareness during simulated VFL compared to NV.

Qualitative analysis of interviews revealed that a vast majority of the participants perceived challenges in hazard detection when driving with simulated VFL compared to NV. Lateral guidance, interaction with other traffic participants, and longitudinal guidance were also reported to be challenging. Only one participant had problems with achieving a general overview of the driving scene (see Figure 4). No differences between vision conditions were evident. Except for situational awareness with low scores for all intersection types, all areas were more frequently rated as challenging for turning maneuvers compared to SCI. Subjective reports on applied compensatory strategies varied in terms of the affected domain, combination of different individual behaviors, and the level of details. In summary, visual adaptations were mentioned most frequently in terms of greater and more repeated gaze movements, particularly toward the blind peripheral field of view as well as a general dislocation of the head and/or eyes toward the blind side. This type of compensatory behavior was mentioned in 65.79% of reported adaptative strategies. The second most common domain was longitudinal adaptation in terms of a general reduction of speed as well as earlier and more frequent braking, which was mentioned in 47.37% of strategies. Other less-frequent mentions covered a dismissal of regulation rules and a general halting at intersections, greater speed when driving through the intersection, longer waiting times, and scanning behavior to try and look past the simulated VFL.

## Discussion

### Driving Behavior and Safety

No overall effects for a deviation of the ideal lane position could be found. The participants generally held a lane position toward the left side in all vision conditions, potentially due to a higher risk estimation for collisions with hazards on the sidewalk or parked vehicles compared to oncoming vehicles. During LHH, the subjects adjusted their lane positions slightly to account for the increased risks of oncoming vehicles on the blind side. Overall, a buffer on the blind side was held in approximately one-third of cases for both LHH and RHH. Previous reports on the lane position offset in VFL and its laterality have been inconsistent (Szlyk et al., 1993; Tant et al., 2002; Wood et al., 2011; Haan et al., 2014). A buffer on the blind side is, however, typically regarded as compensatory behavior, even though a dislocated lane position can also bring an increased risk of collisions on adjacent lanes depending on its extent.

The frequently reported increase in lane position variance as an indicator of poorer steering stability among drivers with VFL (Tant et al., 2002; Bowers et al., 2010; Wood et al., 2011; Haan et al., 2014; Kasneci et al., 2014) was present in our study on a descriptive level with a lower mean and range of variance in the lane position offset during NV. Overall, lane crossings as a measure of driving safety (Bowers et al., 2010) during the deceleration phase were seldom but solely apparent in drives with simulated VFL. This accentuates the tendency for increased difficulties concerning steering stability and lane keeping with an altered lateral positioning under VFL, whereby some drivers hold a buffer on the blind side and fewer others on the seeing side.

In accordance with these results, subjective safety was rated to be significantly lower for drives with simulated VFL compared to NV. This is also in line with the notion that vision is crucial for safe driving (Owsley and McGwin, 2010) and on-road as well as simulator studies, reporting that some drivers with VFL are unfit to drive, albeit percentages range between 23 and 86 (Coeckelbergh et al., 2002; Tant et al., 2002; Elgin et al., 2010; Kasneci et al., 2014).

The second most frequently mentioned strategy to overcome challenges of simulated VFL in the present study was the adjustment of speed and deceleration behavior. Objective data did not reflect these subjective reports since, overall, time from first braking to entering the intersection did not differ between vision conditions. While reported as one of the most prominent compensatory strategies for drivers with central vision loss (Patterson et al., 2019), literature concerning the effect of peripheral VFL on longitudinal guidance is divided. While some have found increased speed levels (Bowers et al., 2009; Wood et al., 2011), others have reported greater speed levels (Bahnemann et al., 2015) or a general heterogeneity among patients (Coeckelbergh et al., 2002). A great heterogeneity of stopping behavior was also evident in the present study, where the distance to the intersection when first braking ranged between almost 100 m before the intersection and no braking at all. While some participants noted speed adjustment as a compensatory strategy, others ranked it as particularly difficult due to the missing feedback of the static driving simulator and an improper dislocation of the gaze-contingent mask during downward gazes toward the speedometer.

While overall scenario time as an approach to objectify the perceived complexity of and cautiousness during the deceleration phase did not yield group differences, subjective workload levels evaluated for the entire intersection scenario were found to be significantly higher for simulated VFL. This supports the proposition by Biebl et al. (2021) that visual impairments and the requirement to compensate for them can lead to cognitive overload and, as a result, an increased safety risk. For simulated VFL conditions, turns toward the seeing side received the highest workload scores. When the subjects viewed the turning path in this combination of vision condition and intersection type, the blind visual field covered most of the driving scene. This aggravates maintaining an overview of the other vehicles' behavior and lateral guidance, especially during the turning process that follows the deceleration phase.

### Visual Behavior

Visual behavior was most frequently mentioned as compensatory strategy, which is reflected in many objective parameters of visual behavior. Laterality of the first large peripheral gaze movement showed an intricate pattern. Past research has suggested a tendency to first scan the blind side when approaching an intersection, although the number of participants exhibiting this behavior varied (Alberti et al., 2014; Bowers et al., 2014). In the present study, most participants first scanned the right hemifield when approaching a straight intersection, which contradicts the general *look left—look right—look left* rule and the idea of a compensatory first blind-side scan at least for LHH. During the presented analysis, the scans were considered only during the deceleration phase after hitting the brakes, since scanning in the preceding approach phase is less focused on hazard perception and identification (Plavsic, 2010). Since initial brake activation occurred very close to reaching the intersection in some cases, it cannot be ruled out that earlier scans to the left side were made. Different patterns for vision conditions became apparent for turning maneuvers, where most participants first scanned the left periphery with NV, the right periphery with RHH, and the right or left periphery during left and right turns with LHH, respectively. Laterality of the first scans with NV is in line with the *look left—look right—look left* rule and the findings from Bowers et al. (2010). The behavioral change to scan the right periphery first during both turns with RHH and right turns with LHH conforms with the assumption of compensatory first blind-side scans (Bowers et al., 2014; Alberti et al., 2017). More participants scanning the seeing side first instead of the blind side first during left turns with LHH contradicts this conjecture. It should be noted that comparisons between right-sided and left-sided first scans yielded a maximum ratio of 3:2, which means that a non-negligible part of the sample behaved conversely. One explanation for the tendency toward first seeing-side scans only during LTI with LHH could be a context dependence on traffic conditions relevant for safe task performance. Typically, LTI is regarded as more difficult compared to SCI and RTI due to the increased number of potential collision points throughout the visual field (Gerstenberger, 2015). Safe maneuvering, hereby, also requires the identification of a gap to pass through crossing traffic from the left and a gap to turn into and assert the new lane position among crossing traffic from the right. If the latter is assumed to be more difficult than the former, especially with a reduced visual field on either side, a first rightward scan could be a proper behavioral adaptation. Alternatively, one could reason that, since mental demand was highest during LTI with LHH, the otherwise appropriate compensatory first blind-sided scans reached their limit and were not deployed in this condition.

While behavioral changes occurred at least on a descriptive level for most values in the comparison between NH and both simulated VFL conditions, greater challenges with LHH compared to RHH were often evident. Mean gaze eccentricity showed a bias toward the blind side with RHH in all scenarios and, to a smaller extent, with LHH in SCI and RTI compared to the generally leftward mean gaze position with NV. This mirrors reports on a shifted mean gaze eccentricity toward the blind side as part of a compensatory strategy (Coeckelbergh et al., 2002; Papageorgiou et al., 2012; Bowers et al., 2014). During LTI with LHH, the participants held a mean gaze position further to the right compared to NV, which was also a general finding in the comparison of NV and LHH reported by Kübler et al. (2015). Overall, an absence of large gaze movements was more frequently found for simulated VFL and, especially, LHH, with an overall surplus for the right compared to the left periphery.

Overall maximum horizontal dispersion and gaze variance were the smallest during LHH. This supports the notion of increased challenges during LHH compared to RHH (Bahnemann et al., 2015), albeit some authors have not found a difference in horizontal gaze activity under simulated VFL (Kasneci et al., 2014). One closely related parameter that is consistently reported to be increased for simulated and pathological hemianopia is an increased scanning path (Tant et al., 2002; Simpson et al., 2011; Papageorgiou et al., 2012; Nowakowska et al., 2016). However, this parameter might be more indicative for visual search tasks compared to driving.

Typically, larger saccadic amplitudes are associated with better task performance (Hardiess et al., 2010; Papageorgiou et al., 2012; Bahnemann et al., 2015). Studies on pathological and simulated VFL have, however, reported heterogenous findings (Zihl, 1995; Tant et al., 2002), smaller saccadic amplitudes (Pambakian et al., 2000; Tant et al., 2002; Machner et al., 2009) or greater saccadic amplitudes (Schuett et al., 2009b), with simulated and pathological VFL. In the present study, saccadic amplitudes were lower for both vision conditions but, particularly, for LHH. Furthermore, saccadic amplitudes showed greater amplitudes when directed toward the left compared to the right periphery, as well as a very wide range of values due to differing starting points and the varying number of saccades within one scenario. Results from this hemidirectional analysis were mirrored for hemispace analysis of fixation duration, with longer durations in both vision conditions, but, especially, LHH and greater durations on the right than the left side. Longer fixation durations in VFL match reports from earlier studies on pathological and simulated VFL (Zihl, 1995; Tant et al., 2002). The number of fixations was generally increased for the right compared to the left visual field, with the most fixations on each hemifield found during ipsilateral VFL. During RHH, the participants showed more fixations on the blind side than the seeing side in approximately one half of all scenarios. This was less often the case during LHH, where more participants favored the seeing side than the blind side. Literature indicates that an increased compensatory scanning of the blind side is frequently found among real and simulated VFL (Simpson et al., 2011; Wood et al., 2011; Papageorgiou et al., 2012; Bahnemann et al., 2015).

In summary, behavioral changes under simulated VFL could be found with more severe effects and fewer indicators for the adoption of compensatory strategies during LHH compared to RHH. Furthermore, scanning performance was more prone to be neglected or aggravated in and toward the right than the left hemifield. One potential explanation for that is the differing useful field of view of the driving scene. The static driving simulator mock-up enables a view from the driver's seat as experienced during real driving with the vehicle's carrosserie blocking part of the field of view. The left side of an intersection can be seen quite clearly through the front and side window, with only the a-pillar blocking a small part of the visual field. On the contrary, the right hemifield with the identical maximum eccentricity of 90° is blocked by larger parts of the vehicle's carrosserie and a smaller view out of the side window. Due to the smaller useful field of view, scanning the right side for potentially arising hazards might have been aggravated. This could have been especially challenging for LHH, since scans toward the right periphery with this type of simulated VFL go along with an occlusion of, virtually, the entire driving scene display, with only a very small visible part remaining in the right periphery. Greater challenges in scenarios that require large seeing side scans are in line with the increased levels of workload reported for turns toward the seeing side compared to the blind side.

### GCD in Driving Simulations

Overall, findings from this study mirrored challenges and compensatory strategies of studies with real and simulated VFL in many aspects, albeit effect sizes were small and many results evident only on a descriptive level. Therefore, the findings, overall, supported the notion that the simulation of VFL with a GCD within the driving simulator is a valuable tool to enrich patient studies on driving with visual impairments. One of the most distinct and rather consistent findings, however, was the vast interpersonal heterogeneity in almost all variables that not only yielded different extents of differences between conditions but also varying individual scanning patterns with contradictory directions (see Figure 3). Such an interpersonal heterogeneity has frequently been reported previously. Nowakowska et al., for example, found that different samples produced quite diverse effects under first exposure with simulated homonymous hemianopia, where some participants were immediately able to compensate well while others could not (Nowakowska et al., 2016, 2019). The same is reported for participants with pathological VFL, where a multitude of studies has differentiated between high and low performers to account for the broad interpersonal heterogeneity (Bowers et al., 2009; Elgin et al., 2010; Hardiess et al., 2010; Wood et al., 2011; Papageorgiou et al., 2012; Haan et al., 2014; Bahnemann et al., 2015; Kübler et al., 2015; Alberti et al., 2017). Glen et al. (2015) already pointed out that, while the experience with (simulated) VFL differs for each person, simulations provide the benefit of enabling within-subject comparisons. An EAB analysis as used in the present study can, therefore, provide valuable information for the individual comparison of behavior under different conditions by using the NV vision condition as the baseline.

Despite the cautious interpretation of our results as promising indicators for the viability of the GCD for VFL simulations in driving simulators, certain differences between simulated and pathological VFL are inevitable. Some of these differences can be regarded as an opportunity to enhance and get deeper insights into the mechanisms behind driving with VFL if considered appropriately. General benefits of GCDs compared to other methods can be found in the introduction of this paper.

#### Deficits and Lesions

First and foremost, simulated VFL differs from pathological impairments in the absence of an actual anatomical or functional deficit within the visual system. Especially VFL arising from brain lesions are often associated with comorbid deficits in the cognitive, visual, or motoric domain or reduced neuroplasticity and connectivity of neuronal pathways. The difference in location, site, and extent of the cortical damage presented in patients and the resulting heterogeneity of the visual and further comorbid impairments often reduces sample size and introduces a great variance within patient samples that diffuses systematic effects. Simulated vision loss enables a homogenization of samples to systematically manipulate certain factors of interest and to get a clearer view of the visual impairments' sole effect on driving performance. When investigating visual exploration with VFL, multiple researchers have pointed out additional higher-order effects like visuo-spatial processing, memory, and organizational functions (Tant et al., 2002; Nowakowska et al., 2016, 2019), as well as cortical plasticity (Glen et al., 2015). It can be assumed that different levels of performance in these cognitive functions also account for the heterogeneity of challenges and behavioral adaptations upon initial exposure with a simulated VFL even in the absence of cortical damages (Tant et al., 2002; Nowakowska et al., 2016, 2019), which was also found in the present study. One particular benefit of simulated vision loss in this context is the ability to quantify the “premorbid” performance that is unknown for pathological VFL in most cases, except for long-term prospective studies. Within-subject comparisons allow for a more refined estimation of the visual impairments' effects in due consideration of interpersonal differences. On an inferential level, this can be done *via* mixed-linear models that include random person effects. The EAB analysis used for a description of the effect of simulated vision loss compared to baseline NV can serve as a useful tool for descriptive analyses. One further problem resulting from the large heterogeneity of driving and visual behavior under simulated VFL is the aggravated validation of simulations, which was already mentioned by Geringswald et al. (2013). Null effects, therefore, cannot clearly be attributed to unrealistic simulations or random sample effects.

#### Sample Characteristics

Secondary consequences of the cortical damage introduce differing sample characteristics between simulated and pathological VFL. Most patients do not take part in driving simulator studies until 3 or even 6 months have passed since the onset of VFL due to hospitalization, rehabilitation programs or instability that inhibits study participation. For degenerative diseases, the time since onset might increase even more due to not being aware of the disease in its early stages. Participants with simulated hemianopia, on the other hand, are tested immediately upon exposure, which provides a different level of expertise and adaptation to the VFL. Simulations, therefore, provide an estimation of the immediate effects of VFL without influence of prior spontaneous or systematically trained compensatory strategies. Furthermore, they allow the observation of this process of strategy development. However, the evaluation of long-term effects of living with and adapting to a pathological VFL over months or years cannot be achieved with simulated VFL. A study on the development of compensatory strategies by Simpson et al. (2011) showed that adaptation to VFL is not linear but, rather, a biphasic process with fast qualitative improvements in the beginning and slower improvements of efficiency later. Even though the initial adaptation seems to proceed faster during simulated VFL (Schuett et al., 2009a), Macnamara et al. (2021) argued that the effects may be aggravated due to missing long-term experience. The present study indicates a mixed picture of effective compensatory strategies in some participants but not others and between LHH and RHH. Upon initial exposure, gaze behavior might also be influenced when applying a GCD since peripheral targets stabilized upon foveal positions can trigger smooth pursuit movements (Aguilar and Castet, 2011). While the displayed mask for the VFL simulation is not a target *per se*, a similar attraction of attention to the gaze-contingent object cannot be ruled out. A prolonged adaptation period can counteract such effects (Aguilar and Castet, 2011). The importance of adequate practice trials with the simulation before measuring behavior to allow for initial familiarization with the VFL has been mentioned frequently (Schuett et al., 2009a; Glen et al., 2015; Nowakowska et al., 2016). However, it must be taken into consideration that extended adaptation periods can enable an unwanted development of compensatory strategies (Macnamara et al., 2021).

#### Applicability

The tremendous relevance of the simulator setup, including spatial and lateral performance, requirements, and limitations of the eye tracker, as well as distance and size of the screen, is already elaborated in the description of the VFL simulation in this paper. Comparability between studies of simulated VFL is, therefore, reduced since lab facilities and algorithms to simulate VFL differ between studies. In our setup, the visual impairment was displayed on large screens surrounding a vehicle mock-up. The rearview and side mirrors and the interior of the vehicle were not covered by the VFL. Tasks with a focus on visual exploration of those parts of the visual scene or any part outside of the vehicle would, therefore, not be viable. Furthermore, some participants reported difficulties viewing the speedometer in the presented study due to corresponding dislocations of the mask in the peripheral but not the foveal field of view, as well as increased signal instability. The desktop mounted eye tracker also had greater signal instability during large scans to the far-left periphery. This limitation of the eye tracker could be an alternative explanation for the increased difficulties found during LHH, which prompts large left-sided scans to compensate for the VFL. In general, using a GCD to simulate VFL requires a lab setting with the respective computer screens or virtual reality glasses. This limits applicability of use cases in other contexts, as well as prolonged exposure to the simulated deficit during everyday activities. While Goodman-Deane et al. (2013) argue that the frustration and social consequences of pathological VFL can only be induced *via* long-term application, Jones et al. (2020) also found a representation of the emotional aspects of VFL during shorter exposition. GCDs could, therefore, also be applied to investigate anxiety-prone situations, which currently reduce patient mobility [see Taylor et al. (2020) for a patient study], or concerns related to future mobility aids and assistive technologies [see Brewer and Kameswaran (2018) for a patient study]. It must, however, be noted that participants with simulated VFL will most likely show a reduced personal and emotional involvement in the studies' topics. Since persons with VFL, such as homonymous hemianopia, are prevented from driving in many jurisdictions, they might be invested in their own driving performance more intensely during driving simulator experiments.

#### Illustration of VFL

One frequently discussed difference between simulated and pathological VFL is its depiction. Macnamara et al. (2021) argued that the very common usage of a dark spot to simulate VFL is not wrong *per se* since some individuals report such a perception of the blind visual field. The majority of patients with scotomas, following age-related macular degeneration and glaucoma, however, describe their VFL as blurred or missing parts where objects seemingly disappear due to filling-in (Hoste, 2003; Crabb et al., 2013; Lee et al., 2016; Taylor et al., 2018). On the contrary, the frequently used pictural presentations of black or gray patches are dismissed quite consistently (Crabb et al., 2013; Taylor et al., 2018). Wu et al. (2018) tried to address this issue in their central scotoma simulation in a head-mounted display by using a dark spot as absolute scotoma and with blurring on its edges as relative scotoma. There is currently little literature describing the perception of hemianopia or other large peripheral VFLs. The anosognosia concerning their VFL in some patients with hemianopia could result from filling-in (Hoste, 2003; Crabb et al., 2013; Lee et al., 2016). Macnamara et al. (2021), therefore, recommend using a blank VFL, which, however, is not entirely applicable for the dynamic changes in color and luminance within a driving scene. Glen et al. (2015) opted for a blurring of the visual scene in their hazard perception test. However, a blurring might not be entirely sufficient to simulate hemianopia since rough contours and movements of potential hazards could still be noticeable. The blurred transition of a gray overlay with a similar color and luminance to the road surface and surrounding housing in the present study was a mixture of different approaches. To the best of our knowledge, the often-proposed simulation of filling-in effects has not been implemented in a GCD so far. Duchowski et al. (2004) have argued that the perception of non-perception is very difficult to illustrate. Peli et al. (2020) have proposed the usage of seam carving to process static images for scotomas. An application for video-based formats has, however, not been presented at this point. In addition to filling-in or seam carving effects, simulations should further depict properties of the visual system, including the complex interplay between light sensitivity, color and movement perception, and sensitivity to different frequencies (Crabb et al., 2013). Up to 70% of patients have residual visual abilities in their blind field called blindsight, where general low spatial frequencies, such as presence, position, orientation, movement of stimuli, and socially relevant cues, can be perceived in the blind field (Nowakowska et al., 2016). Lee et al. (2016) added that simulations should encompass intraindividual differences of VFL, since the perceived deficit depends on the mental state of mind or on the lighting conditions. The integration of such features not only requires an intricate understanding of the visual system and the perception of VFL for different subjects but also advanced graphical processing software to incorporate respective features. Such an algorithm is surely advisable to enhance comparability with pathological VFL, albeit difficult to implement in driving simulator software. Despite these limitations regarding illustration of the VFL, many studies, including the one presented here, have shown promising effects for simulations to induce similar challenges and compensations found among patients when confronted with a VFL.

#### Suitable Fields of Application

In summary, simulations of VFL and, especially, GCD scan serve as a valuable addition to research on patients with pathological VFL by introducing certain methodological enhancement. More precisely, GCDs allow for the evaluation of a carefully selected and homogenous visual field loss among participants. In that, the utilization of GCDs can help to identify underlying mechanisms that might, otherwise, be occluded by comorbidities accompanying brain injuries. They, furthermore, enable the observation of the effects of VFL immediately upon initial confrontation, as well as the development of compensatory strategies.

It must, however, be noted that the above-mentioned differences produce an inevitable difference between simulated and pathological visual impairments. It is, therefore, not advisable to solely base research on the experience of otherwise healthy participants with temporary visual field occlusions. Instead, the potential of simulated VFL should be exploited to enhance and complement the indispensable examination of actual patients. In this regard, simulated VFL can also be used to test study protocols to save limited resources for patient studies. Lastly, it should be noted that GCDs do not enable a simulation of visual field loss in real traffic since the adoption in on-road tests *via* augmented reality is not safe or ethical. Driving simulators allow for the introduction of potentially risky situations with the use of visual deficits in normally sighted drivers. Other main benefits of driving simulators encompass the ease of data collection and the enabling of controllable, reproducible, and standardized conditions in all drives for all participants (Winter et al., 2012). Overall, the validity of driving simulators to predict on-road driving performance has been indicated (Lee, 2003; Shechtman et al., 2009; Casutt et al., 2014). Nevertheless, all driving simulators have somewhat limited physical, perceptual, and behavioral fidelity, and artifacts, such as simulator discomfort, can arise (Winter et al., 2012). Simulations of VFL with the gaze-contingent paradigm in a driving simulator must be interpreted with respective caution.

### Limitations

To the author's knowledge, this study presents one of the first attempts to simulate VFL in a driving simulator with a 180° frontal field of view, using the GCD paradigm. The associated free movement of eye, head, and torso comes with challenges for the eye tracking system. Obstruction of the eye due to glasses, large head rotations, or certain hand-positions and system latencies challenged eye tracking performance and, as such, VFL continuity. Further iterations of the algorithm should encounter these limitations. It should be noted that the presented VFL depicted complete homonymous hemianopia, which represents a rather severe visual deficit. Another limitation is the small total sample size with an overall young age and potential behavioral changes due to simulator sickness. The aim of this study was to evaluate the comparability of simulated VFL in a driving simulator with pathological VFL. Studies that served as a reference for the effects of pathological VFL have, however, used different methodologies concerning research focus, sample characteristics, tasks, and setup, which introduce an arbitrary variance. To account for the interindividual heterogeneity exhibited by the participants, an individual analysis was performed to identify behavioral changes under simulated VFL compared to a baseline condition with normal vision. However, this approach has not been validated so far and could be the focus of future research.

## Conclusion

In the present study, normally sighted participants drove through three types of intersection (continuing straight, turning left, turning right), with normal vision and right-sided and left-sided simulated homonymous hemianopia. We found reduced subjective safety ratings and a tendency for more lane crossings for drives with simulated VFL. While large interindividual differences were apparent, visual parameters showed an altered behavior under VFL with greater challenges under simulated VFL on the left side and a disparity between scanning of the right and left hemifields. Overall, the findings support the notion that GCDs can enhance research on visual impairments in driving simulator studies to quantify the sole effect of VFL on driving performance and to systematically identify factors determining the ability to compensate for visual field loss.

## Data Availability Statement

The raw data supporting the conclusions of this article will be made available by the authors, without undue reservation.

## Ethics Statement

The studies involving human participants were reviewed and approved by Ethics Committee of the Technical University of Munich. The patients/participants provided their written informed consent to participate in this study.

## Author Contributions

BB conceptualized the study and contributed to data curation, methodology, validation, writing the original draft, and project administration. BB, EA, and SK contributed to software. BB and EA contributed to formal analysis and investigation. BB and KB contributed to resources. BB, EA, SK, JR, and KB contributed to writing, reviewing, and editing the manuscript. BB and JR contributed to visualization. KB contributed to supervision and funding acquisition. All authors have read and agreed to the published version of the manuscript.

## Funding

This work was funded by the Deutsche Forschungsgemeinschaft (DFG) under Grant No. BE4532/15-1 to KB and Grant No. RI1511/3-1 to JR (Learning from Humans-Building for Humans) and PR1266/3-1 to AP (Design Paradigms for Societal-Scale Cyber-Physical Systems).

## Conflict of Interest

The authors declare that the research was conducted in the absence of any commercial or financial relationships that could be construed as a potential conflict of interest.

## Publisher's Note

All claims expressed in this article are solely those of the authors and do not necessarily represent those of their affiliated organizations, or those of the publisher, the editors and the reviewers. Any product that may be evaluated in this article, or claim that may be made by its manufacturer, is not guaranteed or endorsed by the publisher.

## Abbreviations

GCD: Gaze-contingent display
VFL: Visual field loss
NV: Normal vision
LHH: (simulated) Left-sided homonymous hemianopia
RHH: (simulated) Right-sided homonymous hemianopia
SCI: The intersections where the participants had to cross/go straight
LTI: The intersections where the participants had to turn left
RTI: The intersections where the participants had to turn right.
