# Supplementary material for: Opportunities and Limitations of a Gaze-Contingent Display to Simulate Visual Field Loss in Driving Simulator Studies
Source: Front Neuroergon. 2022 Jun 10;3:916169. doi: 10.3389/fnrgo.2022.916169 (PMC10790882; doi:10.3389/fnrgo.2022.916169)
Supplement: Supplementary file 1 [file Data_Sheet_1.PDF]

Supplementary Material

Supplementary Tables 1-6. The following tables contain a comprehensive presentation of relevant descriptive values that were not mentioned in the main text; *M*=mean; *SD*=standard deviation; *Above*=percentage of participants with higher values compared to intraindividual normal vision values; *Below*=percentage of participants with lower values compared to intraindividual normal vision values; *Equal*=percentage of participants with equal values compared to intraindividual normal vision values; *Miss*=no fixation/saccade in the simulated visual field loss or normal vision condition

Parameter	Value	Condition								
		SCI			RTI			LTI		
		NV	LHH	RHH	NV	LHH	RHH	NV	LHH	RHH
**Subjective workload (0-20)**	*M*	5.87	10.60	11.27	8.08	14.27	10.67	8.27	12.07	13.00
	SD	3.48	3.91	4.70	3.96	5.38	4.20	4.54	4.11	5.54
	Above		86.67%	80.00%		75.00%	25.00%		60.00%	66.67%
	Below		0.00%	13.33%		0.00%	8.33%		13.33%	6.67%
	Equal		13.33%	6.67%		25.00%	66.67%		26.67%	26.67%
**Scenario duration (sec)**	*M*	7.38	8.54	8.38	7.75	7.76	7.92	8.21	7.73	9.46
	SD	3.65	4.94	3.33	3.53	2.89	3.04	3.27	4.73	3.85
	Above		40.00%	20.00%		25.00%	8.33%		20.00%	46.67%
	Below		20.00%	13.33%		16.67%	8.33%		26.67%	13.33%
	Equal		40.00%	66.67%		58.33%	83.33%		53.33%	40.00%
**Mean lateral lane offset (cm)**	*M*	-15.51	-10.67	-5.10	-18.54	-6.85	-22.05	-13.03	-4.54	-20.69
	SD	36.37	34.38	35.90	29.89	22.04	38.79	27.91	28.05	31.58
	Above		26.67%	33.33%		50.00%	50.00%		26.67%	20.00%
	Below		13.33%	20.00%		16.67%	25.00%		6.67%	33.33%
	Equal		60.00%	46.67%		33.33%	25.00%		66.67%	46.67%

Parameter	Value	Condition								
		SCI			RTI			LTI		
		NV	LHH	RHH	NV	LHH	RHH	NV	LHH	RHH
**Variance lateral lane offset (cm)**	*M*	0.89	3.67	3.19	0.67	1.51	1.94	0.48	1.17	1.36
	SD	1.00	6.73	9.70	0.68	2.21	2.04	0.39	2.29	1.17
	Above		33.33%	13.33%		33.33%	66.67%		33.33%	60.00%
	Below		40.00%	33.33%		25.00%	16.67%		26.67%	20.00%
	Equal		26.67%	53.33%		41.67%	16.67%		40.00%	20.00%
**Mean gaze Eccentricity (°)**	*M*	0.33	-0.93	4.82	-6.41	-8.36	-0.27	-2.18	3.51	5.54
	SD	7.06	15.51	11.14	8.31	17.93	15.81	8.84	8.53	15.64
	Above		26.67%	53.33%		33.33%	41.67%		53.33%	53.33%
	Below		46.67%	20.00%		50.00%	25.00%		20.00%	20.00%
	Equal		26.67%	26.67%		16.67%	33.33%		26.67%	26.67%
**Horizontal Variance Gaze (°)**	*M*	975.53	712.81	981.85	823.51	640.19	717.98	916.61	593.16	918.62
	SD	560.8	483.19	541.94	306.46	499.39	421	561.19	391.59	537.33
	Above		13.33%	26.67%		8.33%	8.33%		6.67%	6.67%
	Below		33.33%	20.00%		50.00%	58.33%		40.00%	0.00%
	Equal		53.33%	53.33%		41.67%	33.33%		53.33%	93.33%

Parameter	Value	Condition								
		SCI			RTI			LTI		
		NV	LHH	RHH	NV	LHH	RHH	NV	LHH	RHH
**Min gaze eccentricity (°)**	*M*	-50.82	-42.01	-50.05	-63.26	-45.70	-52.85	-53.08	-44.19	-54.52
	SD	20.28	25.63	23.20	3.02	25.59	19.37	21.28	23.29	21.37
	Above		40.00%	33.33%		75.00%	75.00%		46.67%	20.00%
	Below		13.33%	26.67%		16.67%	8.33%		13.33%	33.33%
	Equal		46.67%	40.00%		8.33%	16.67%		40.00%	46.67%
**Max gaze eccentricity (°)**	*M*	54.70	45.33	55.01	42.32	36.68	42.31	53.56	45.70	53.34
	SD	16.80	25.13	20.12	22.73	22.42	22.68	19.02	22.59	18.20
	Above		6.67%	20.00%		0.00%	8.33%		13.33%	26.67%
	Below		46.67%	26.67%		33.33%	8.33%		40.00%	13.33%
	Equal		46.67%	53.33%		66.67%	83.33%		46.67%	60.00%

Parameter	Value	Condition								
		SCI			RTI			LTI		
		NV	LHH	RHH	NV	LHH	RHH	NV	LHH	RHH
Mean number fixation	**Left hemifield**									
	M	2.07	4.40	2.40	3.25	4.47	2.47	2.93	2.13	2.80
	SD	2.09	4.39	2.38	3.60	3.74	3.78	3.08	2.23	3.32
	Above		40.00%	20.00%		66.67%	16.67%		33.33%	13.33%
	Below		20.00%	13.33%		16.67%	41.67%		40.00%	20.00%
	Equal		40.00%	66.67%		16.67%	41.67%		26.67%	66.67%
	**Right hemifield**									
	M	5.20	4.27	5.93	3.58	3.60	4.40	3.13	4.07	5.53
	SD	4.30	3.71	4.32	3.15	2.77	4.29	2.03	3.20	4.88
	Above		20.00%	26.67%		16.67%	50.00%		40.00%	60.00%
	Below		33.33%	26.67%		33.33%	25.00%		20.00%	20.00%
	Equal		46.67%	46.67%		50.00%	25.00%		40.00%	20.00%

Parameter	Value	SCI NV	SCI LHH	SCI RHH	RTI NV	RTI LHH	RTI RHH	LTI NV	LTI LHH	LTI RHH
Mean duration fixations (ms)	**Left hemifield**									
	M	249.98	282.96	299.40	251.39	296.19	294.62	255.42	305.71	317.12
	SD	115.67	95.77	141.26	130.65	112.89	103.20	98.59	191.54	107.95
	Above		33.33%	26.67%		41.67%	33.33%		6.67%	33.33%
	Below		20.00%	13.33%		8.33%	16.67%		13.33%	20.00%
	Equal		13.33%	20.00%		13.33%	6.67%		26.67%	20.00%
	Miss		33.33%	40.00%		36.67%	43.33%		53.33%	26.67%
	**Right hemifield**									
	M	251.72	340.18	297.25	270.43	387.97	300.39	308.43	365.82	317.35
	SD	81.17	182.89	113.06	128.36	247.72	111.08	141.18	181.96	137.55
	Above		33.33%	46.67%		41.67%	41.67%		40.00%	26.67%
	Below		13.33%	6.67%		0.00%	25.00%		26.67%	26.67%
	Equal		26.67%	33.33%		26.67%	6.67%		6.67%	33.33%
	Miss		26.67%	13.33%		31.67%	26.67%		26.67%	13.33%

Parameter	Value	Condition								
		SCI			RTI			LTI		
		NV	LHH	RHH	NV	LHH	RHH	NV	LHH	RHH
Mean amplitude saccades (°)	**Left hemifield**									
	M	31.03	18.21	26.04	30.61	14.83	26.06	35.70	23.84	32.77
	SD	26.16	15.61	12.45	12.57	7.07	11.11	16.81	15.41	28.39
	Above		6.67%	33.33%		16.67%	33.33%		33.33%	33.33%
	Below		53.33%	46.67%		75.00%	33.33%		40.00%	46.67%
	Equal		13.33%	6.67%		6.67%	26.67%		13.33%	6.67%
	Miss		26.67%	13.33%		1.67%	6.67%		13.33%	13.33%
	**Right hemifield**									
	M	29.51	23.60	21.98	24.71	20.95	22.73	25.47	18.76	28.43
	SD	14.81	10.00	11.58	12.41	17.21	22.56	13.47	10.29	26.29
	Above		6.67%	13.33%		0.00%	0.00%		20.00%	20.00%
	Below		33.33%	26.67%		25.00%	50.00%		40.00%	33.33%
	Equal		26.67%	40.00%		33.33%	26.67%		26.67%	40.00%
	Miss		33.33%	20.00%		41.67%	23.33%		13.33%	6.67%
